# A formative cross-sectional study to assess caregiver’s health-seeking behaviour and knowledge surrounding malaria, and understand the burden of malaria among children under-five in conflict-affected communities of Cameroon

**DOI:** 10.1186/s12936-024-04902-5

**Published:** 2024-04-08

**Authors:** Elvis Asangbeng Tanue, Lundi-Anne Omam, Glennis T. Ayuk, Bibiche Modjenpa Noukeme, Alain Metuge, Isabelle Nganmou, Margaret Besem Ebob, Laura Donovan, Ann-Sophie Stratil, Helen Counihan, Claude Ngwayu Nkfusai, Helen Hawkings, Blanka Homolova, Elizabeth Berryman, Maxwell Kolawole, Yakouba Zoungrana, Dorothy Achu, Samuel Wanji, Esther Njomo Omam

**Affiliations:** 1 Reach Out Cameroon, Small Soppo, P.O Box 88, Buea, Cameroon; 2 Department of Public Health and Hygiene, Faculty of Health Sciences, University of Buea, P.O Box 63, Buea, Cameroon; 3 Department of Public Health and Primary Care, University of Cambridge, Cambridge, CB2 0SR UK; 4 Konmofamba Actions Sans Frontieres, Penja, Cameroon; 5 Malaria Consortium, the Green House, 244-254 Cambridge Heath Rd, London, E2 9DA UK; 6 Department of Public Health, School of Nursing and Public Health, University of Kwa-Zulu Natal, Durban, South Africa; 7 National Malaria Control Programme, Ministry of Public Health, P.O Box 14386, Yaounde, Cameroon; 8 Department for Microbiology and Parasitology, University of Buea, P.O Box 12, Buea, Cameroon; 9 Research Foundation in Tropical Diseases and Environment, Buea, Cameroon

**Keywords:** Malaria, Knowledge, Attitudes, Practices, Health-seeking, Conflicted-affected, Cameroon

## Abstract

**Background:**

Malaria remains a major global health problem often worsened by political instability and armed conflict. The purpose of the study was to explore community knowledge, attitudes and practices on malaria prevention, and to understand the burden of malaria and health-seeking behaviours of caregivers of children under-five in conflict-affected communities of the South West and Littoral Regions of Cameroon.

**Methods:**

A cross-sectional survey involving internally displaced persons (IDPS), host population, and their children under-five was conducted across 80 communities. The survey was conducted from May to June 2021. Participants were interviewed using a structured questionnaire. Malaria prevalence for children under-five was determined using rapid diagnostic tests (RDT) on blood samples. Association between variables and displacement status was measured using chi square test and multivariate logistic regression model was fitted to identify factors associated with adequate knowledge on malaria prevention.

**Results:**

A total of 2386 adults participated in the study and 1543 RDTs were conducted for children under-five. Adequate levels of knowledge and attitudes on malaria prevention was recorded among 1258 (52.9%) of the participants, with very strong evidence to suggest the level to be higher among the host (59.5%) compared to the IDPs (49.5%) and returnees (39.7%) (p < 0.001). Good practices towards malaria prevention was 43.3%, with very strong evidence indicating lower levels among IDPs (42.8%) and returnees (28.5%) compared to the host (49.4%) (p < 0.001). Malaria prevalence for children under-five was 54.0% and adequate health-seeking for suspected episodes of malaria was 53.0%, without any difference among IDPs (51.78%) and returnees (48.7%) compared to host populations (55.4%) (p = 0.154). Multivariate logistic regression model showed that there was quite strong evidence to suggest primary and secondary levels of education have higher odds of having correct knowledge of malaria prevention (adjusted odds ratio (AOR) 1.71, 95% confidence interval (CI): 1.11–2.64, p = 0.015 and AOR 1.80, 95% CI 1.15–2.82, p = 0.010 respectively). There was very strong evidence to suggest that owning a radio or a television was associated with greater odds of having a higher knowledge on malaria prevention (AOR 1.49, 95% CI 1.233–1.81, p = 0.000 and AOR 1.47, 95% CI 1.18–1.84, p = 0.001).

**Conclusion:**

Over half of the population have correct knowledge and attitudes towards malaria prevention but gaps in complete knowledge remained. Some of the caregivers know the correct malaria preventive practices coupled with largely unsatisfactory treatment approaches and reflected by the high prevalence of malaria among their children. In order to effectively treat malaria, innovative strategies should target community participation.

**Supplementary Information:**

The online version contains supplementary material available at 10.1186/s12936-024-04902-5.

## Background

Malaria is a preventable and curable deadly disease. Almost half of the world’s population are at risk [1]. In 2021, 247 million cases of malaria were reported worldwide [1]. Sub-Saharan Africa bears most of the global burden of malaria cases (95%) and 96% of all deaths [1]. It has been estimated that 78.9% of those deaths occurred in children under five [2]. Malaria is an important health problem within conflict-affected contexts, where disease risk factors are enhanced [3]. Armed conflicts pose various direct and indirect challenges to public health. [4]. People are forced to be displaced and resettle in areas with worse environmental and housing conditions. These displacements are usually accompanied by limited and disrupted national malaria control programme together with insecurity resulting in the breakdown of primary healthcare systems impeding access to quality and timely delivery of medical care [5, 6]. Frequent displacement resulting to less secure housing and inaccessibility to insecticide-treated bed nets exposes people to mosquitoes and may increase vulnerability to malaria in a relatively high-risk population.

The South West (SW) and North West (NW) Regions of Cameroon have been facing armed-conflict that has resulted in a humanitarian crisis following the displacement of over 630,000 people into different localities within the country and more than 86,000 people to Nigeria [7–9]. This crisis has led to disruptions in the functionality of the primary healthcare system [8], which has consequently impacted on the quantity and quality of malaria related data in some of the most affected areas. As of April 2023, approximately 18.0% of health facilities have been forced to close, and a number of operational facilities are struggling to function adequately [7], limiting access to health care and malaria prevention/treatment services in particular.

According to the 2018 Demographic and Health Survey (DHS) in Cameroon, malaria prevalence among children under-five experienced a decrease from 30.0% in 2011 to 24.0% in 2018. In the SW and Littoral regions, the malaria prevalence in children under-five pre-conflict was 9.8% and 20.6%, respectively. [10]. The SW Region has the lowest insecticide-treated net coverage in the country, with only 51.0% of the households being able to access them [10]. However, only urban areas entered the 2018 DHS due to insecurity concerns, and the coverage percentage is expected to be even lower overall [10]. More so, data from the 2018 DHS indicate that only 21% of children under-five with a fever or positive malaria rapid diagnostic test (RDT) have access to artemisinin-based combination therapy [10]. According to the World Malaria report, a fever prevalence of 16.0% was reported from the household survey conducted in 2018 [2]. The World Health Organization (WHO) estimates that 6,665,957 cases of malaria have been detected in Cameroon in 2021, resulting in 13,839 deaths [2]. The real prevalence of malaria in children under-five in conflict-affected communities is still unknown. The burden of malaria in conflict-affected settings could be reduced with carefully designed, context-specific interventions [8]. To inform the selection and design of an effective approach to break barriers that prevent people from adopting malaria prevention and control strategies, and improve access to community-based effective malaria treatment, Reach Out in collaboration with Malaria Consortium and “Konmofamba Action Sans Frontières”, conducted a formative observational study to explore the knowledge and attitude gaps of the population, better understand the burden of malaria in children under five and the healthcare-seeking behaviours of caregivers for episodes of the disease in conflict-affected communities of the SW and Littoral Regions of Cameroon.

## Methods

### Study design

A cross-sectional study was conducted between May and June 2021 to obtain baseline data for the context-specific design of interventions to facilitate access to effective malaria treatment. Community knowledge, attitudes and practices on malaria prevention and caregiver’s health-seeking behaviours for suspected episodes of malaria among under-five years children were recorded using structured questionnaires. Malaria prevalence in under-five years children was determined by conducting malaria rapid diagnostic tests (RDT) (SD Biosensor Inc.).

### Study area

Two conflict-affected regions of Cameroon, the SW (ongoing conflict) and Littoral (communities hosting IDPs) were purposefully selected for the study due to scarcity of health data and presence of IDPs. The study was conducted across 80 communities constituted by a single village or group of villages. In the SW Region, the health districts of Ekondo-Titi, Muyuka, Tombel, Kumba-South and Kumba-North were selected for the survey. A total of 16 communities were selected from each of these health districts while the Kumba-North and Kumba-South Health Districts jointly contributed 16 communities. In the Littoral Region, the study included 16 communities chosen from four health districts (Nkongsamba, Mbanga, Melong and Manjo) across the Mongo division, where the IDPs live in peri-urban and urban areas. The health districts in the SW are characterized by frequent restricted movements (or lockdowns), abductions, targeted kidnapping and active fighting between non-state armed groups and government forces [8, 9]. Public services are disrupted and some health facilities have been damaged while health staff have abandoned health centres to seek refuge in safer localities [8]. The IDP populations live in hard-to-reach areas with geographical inaccessibility and financial barriers to health services. Health areas were selected through convenience sampling in consultation with district health authorities to ensure a relevant IDP population was accessible.

### Study population

The study population was composed of four groups of participants: the adult members of the community (resident in the study area before the onset of the conflict), returnees (adults who were displaced due to conflict but had returned), IDPs (present in the study area as a consequence of conflict displacement) and under-five year children in the study communities.

### Inclusion and exclusion criteria

Adults of both sexes resident in the conflict-affected localities, and displaced adults living in the host communities and who were willing to provide informed consent, were included in the study. Individuals with disabilities that prevented them from understanding the questionnaire questions were excluded from this study. Consent was obtained from caregivers of under-five children irrespective of history of fever and a finger prick was performed for RDT for all children under-five in the household.

### Sample size determination

The sample size was calculated so that it could capture changes in “proportion of IDP community members who were aware of appropriate health seeking behaviour for suspected malaria” before and after the project interventions. A sample size of 672 individuals at baseline and endline timepoints was calculated to be sufficient to have 80% power to detect a 10% increase in the primary outcome at 5% significance level, assuming a design effect of 1.5, and a non-response rate of 10%.

To achieve a precise estimate (± 2.5%) of the secondary outcome “RDT-measured malaria prevalence among under- five years children” we assumed that the number of under-five years children in the target population was 145,584 across both regions, that the malaria prevalence was 13%, and 10% of data loss. After factoring in a design effect of 1.5, it was calculated that 1,142 children under-five years would be needed at baseline and endline, to detect differences before and after the interventions at 95% confidence levels.

### Sampling and recruitment process

After all accessible health areas in the study area were identified, communities included in the study were randomly selected by pulling 16 concealed pieces of paper with the community names. At the community level, households were purposefully chosen from areas that were safe and accessible to Community Health Workers (CHWs) until sample size was reached. The KAP survey was administered to one adult member from each selected household. All under-five children from households sampled for the KAP survey were included in the malariometric component of the survey. In each study community, the IDPs were identified according to their settlement.

### Data collection

A paper-based structured closed-ended questionnaire was used to collect information about knowledge, attitudes and practices related to malaria prevention and health seeking behaviour for episodes of suspected malaria (Additional file 1). Trained facilitators and research assistants administered the surveys in English and French when necessary. The questionnaire included questions to evaluate the knowledge on the transmission of malaria, knowledge on methods of malaria prevention, possible symptoms of malaria, seeking help when any malaria symptom is present, or knowledge on the name of the drugs used in the management of malaria, amongst others. The questionnaire included a section to capture suspected caregiver-reported malaria cases in children during the previous 2 weeks. Questions with a single correct response were scored 1 while questions with multiple correct responses were weighted and scored according to the number of right answers to sum up to 1. Before implementation, a pre-test was completed by the project research team with a sample of study participants (that were not included in the final analysis). The survey tool was subsequently adapted to be more appropriate, culturally sensible and comprehensible for the specific context. Quality control procedures during field work included daily checks by supervisors, which consisted of observing at least one interview per enumerator per day and reviewing all completed questionnaires.

### Data transfer and storage

Paper questionnaires and consent forms were always stored in opaque carriers. At the end of data collection, the paper data forms and the consent forms were transferred to the project office by Field Supervisors for storage in a safe locker. Access to the data was restricted to only the data analyst and the Operational Research Specialist.

### Data analysis

Data validation and quality control was done by double entry and data management was done using *Epi-Info* (Version 7.2.4.0) software. A national research consultant supervised the data entry process and validated datasets on a daily basis. The validated datasets were subsequently cleaned, merged, and analysed using the Statistical Package for Social Sciences (SPSS) software version 25.0. The mean score recorded for the knowledge and attitude questions was calculated and participants who scored below the mean value were considered to have an inadequate knowledge and attitude, while participants who scored the mean value and above were considered to have an adequate level of knowledge and attitude towards malaria prevention. Association between variables was exploited using the chi-square test of independence and the enter method of the multivariate logistic regression model was utilized to explore the relationship between socio-demographic variables and the knowledge and attitudes of community members towards malaria prevention. Point prevalence of malaria among children under 5 years old was calculated as children with a positive RDT result among all children that were tested. Malaria positive children were segregated into symptomatic (presented with fever 2 weeks prior to the survey) and asymptomatic (no reported occurrence of fever two weeks prior to the survey).

### Ethical and regulatory considerations

Ethical approval was obtained from the Institutional Review Board of the Faculty of Health Sciences of the University of Buea (Reference number: 2021/1286–02/UB/SG/IRB/FHS). Administrative authorization was obtained from the South West and Littoral Regional Delegation of Public Health and the District Health Services for all study sites. In alignment with the Declaration of Helsinki, written informed consent was secured from all study participants, including caregivers of children before a malaria RDT was performed. Participants were informed they had the right to withdraw at any time from the study if they decided. To ensure participants’ confidentiality, the questionnaires were anonymized by assigning unique codes to each participant.

## Results

### Socio-demographic characteristics of community members

A total of 2,386 participants took part in the study. Table 1 shows the socio-demographic characteristics of the study participants. IDPs constituted 24.0% (n = 572) of the respondents. More than half of the participants were females (1371; 57.5%) and most (687; 28.8%) of the participants were aged between 26 and 35 years. About half of the respondents were household heads (1216; 51.0%) and of the majority (1541; 64.6%) of the participants were self-employed practicing agriculture and small businesses as their main source of income. Respondents with a primary level of education constituted 46.7% (n = 1115) of the study. A good proportion of the community members had access to the radio (1142; 47.9%), television (1519; 63.7%) and owned mobile phones (1979; 82.9%).

**Table 1:** Socio-demographic characteristics of study participants from the conflict-affected communities of the South West and Littoral Regions of Cameroon (n = 2386)

Variable	Category	Study population (column %)
Gender^a^	Male	1012 (42.4)
	Female	1371 (57.5)
Age group (years)^a^	18–25	326 (13.7)
	26–35	687 (28.8)
	36–45	596 (25.0)
	46–55	441 (18.5)
	> 55	327 (13.7)
Religious affiliation^a^	Christian	2261 (94.8)
	Muslim	41 (1.7)
	Traditional belief	60 (2.5)
	Others	12 (0.5)
Relationship with the head of this household	Head of household	1216 (51.0)
	Spouse	710 (29.8)
	Son/daughter	286 (12.0/
	Others	174 (7.3)
Main occupation	Domestic worker	132 (5.5)
	Employed	361 (15.1)
	Unemployed	148 (6.2)
	Others	67 (2.8)
	Self-employed	1541 (64.6)
	Student	137 (5.7)
Highest level of education	Informal	103 (4.3)
	Primary	1115 (46.7)
	Secondary	977 (40.9)
	Tertiary	191 (8.0)
Type of available household assets	Radio	1142 (47.9)
	Television	1519 (63.7)
	DVD player	732 (30.7)
	Mobile phone	1979 (82.9)
	Motor bike	413 (17.3)
Displacement status	Internally displaced persons	572 (24.0)
	Returnees	513 (21.5)
	Host	1301 (54.5)

^a^Denotes variables with missing values

### Community knowledge and attitude towards malaria prevention

The mean knowledge and attitude score towards malaria prevention was 9.14 (SD ± 2.06). Over half (1258; 52.9% (95% CI 50.9%-54.9%)) of the community members had an adequate level of knowledge and attitudes on malaria prevention (Table 2). The level of knowledge and attitudes towards malaria prevention was higher among the host population (774; 59.5%) compared to the IDPs (282, 49.5%) and returnees (202; 39.7%) (p < 0.001). The host population compared to the returnees and IDPs, had better knowledge concerning malaria transmitted by mosquito bites (p = 0.001), avoiding outdoors at night in order to prevent malaria (p < 0.001) and symptoms of malaria, including fever (p < 0.001), headache (p < 0.001), body aches (p < 0.001) and nausea (p < 0.001).

**Table 2:** Knowledge and attitude concerning various malaria aspects of community members in the conflict-affected settings of the South West and Littoral Regions of Cameroon

Variable	Category	Total	Status of study population			p-value^*a*^
			IDPs No (%)	Returnee No (%)	Host No (%)	
Malaria transmission	Mosquito bite	2271	536 (94.0)	475 (93.3)	1260 (96.9)	0.001
	Others	108	34 (6.0)	34 (6.7)	40 (3.1)	
Methods to avoid getting malaria	Use a mosquito net	2197	516 (90.5)	459 (90.2)	1222 (94.0)	0.004
	Avoid outdoors at night time	804	235 (41.2)	147 (28.9)	623 (47.9)	< 0.001
	Other correct answers	261	56 (9.8)	56 (11.0)	149 (11.5)	0.580
Malaria symptoms	Fever	2252	537 (94.2)	464 (91.2)	1251 (96.2)	< 0.001
	Headache	1616	385 (67.5)	269 (52.8)	962 (74.0)	< 0.001
	Body aches	787	174 (30.5)	134 (26.3)	479 (36.8)	< 0.001
	Blood in urine	188	49 (8.6)	42 (8.3)	97 (7.5)	0.667
	Diarrhoea	233	87 (15.3)	41 (8.1)	105 (8.1)	< 0.001
	Nausea/vomiting	922	215 (37.7)	147 (28.9)	560 (43.1)	< 0.001
Seek help when any symptom is present	Yes	2291	545 (95.6)	474 (93.1)	1272 (97.8)	< 0.001
	No	78	25 (4.4)	35 (6.9)	28 (2.2)	
Biomedical health-seeking contact	Community health worker	1437	332 (58.2)	296 (58.2)	809 (62.2)	0.135
	Health facility	1372	307 (53.9)	228 (44.8)	837 (64.4)	< 0.001
Knows RDT as diagnostic test	Yes	1147	253 (44.4)	204 (40.1)	690 (53.1)	< 0.001
	No	1232	317 (55.6)	305 (59.9)	610 (46.9)	
Request for an RDT before treatment	Yes	1494	339 (59.5)	269 (52.8)	886 (68.2)	< 0.001
	No	885	231 (40.5)	240 (47.2)	414 (31.8)	
Knows name of drug to treat malaria	Yes	1627	394 (69.1)	300 (58.9)	933 (71.8)	< 0.001
	No	752	176 (30.9)	209 (41.1)	367 (28.2)	
Agree with the following						
Household is affected by malaria	Yes	1743	424 (74.4)	358 (70.3)	961 (73.9)	0.236
	No	636	146 (25.6)	151 (29.7)	339 (26.1)	
Malaria is a matter of concern for me	Yes	2119	499 (87.5)	437 (85.9)	1183 (91.0)	0.003
	No	260	71 (12.5)	72 (14.1)	117 (9.0)	
Malaria can have long-term consequences health	Yes	2046	473 (83.0)	435 (85.5)	1138 (87.5)	0.030
	No	333	97 (17.0)	74 (14.5)	162 (12.5)	
Testing for malaria before treatment	Yes	2228	534 (91.9)	476 (93.5)	1228 (94.5)	0.117
	No	151	46 (8.1)	33 (6.5)	72 (5.5)	
Coartem is the best cure for malaria	Yes	1389	320 (56.1)	272 (53.4)	797 (61.3)	0.004
	No	990	250 (43.9)	237 (46.8)	503 (38.7)	
Knowledge category	Inadequate	1121	288 (50.5)	307 (60.3)	526 (40.5)	< 0.001
	Adequate	1258	282 (49.5)	202 (39.7)	774 (59.5)	

^*a*^*p*-values obtained from the chi-square test of independence

### Malaria prevention practices among community members in conflict-affected settings

The mean malaria practice score was 1.30 (SD ± 0.43). Malaria preventive practices were generally poor with less than half (1031; 43.3%, 95% CI 41.3–45.3%) of the community members presenting with a good level of practice towards malaria prevention (Table 3). The level of good practices towards malaria prevention was found to be lower among the IDPs (244; 42.8%) and returnees (145; 28.5%) compared to the host population (642; 49.4%) (p < 0.001). Furthermore, a higher proportion (1157; 89.0%) of members of the host population practice sleeping under a mosquito bed net than their IDP (455; 79.8%) and returnee counterparts (409; 80.4%) (p < 0.001).

**Table 3:** Practices towards malaria prevention among community members in the conflict-affected settings of the South West and Littoral Regions of Cameroon

Variable	Category	Total	Study population			p-value^*a*^
			IDPs No (%)	Returnee No (%)	Host No (%)	
Household with malaria protective method	Yes	1389	519 (91.1)	447 (87.8)	1226 (94.3)	< 0.001
	No	990	51 (8.9)	62 (12.2)	74 (5.7)	
Protective methods against malaria	Claims to sleep under a mosquito net	2021	455 (79.8)	409 (80.4)	1157 (89.0)	< 0.001
	Use netting on windows	725	137 (24.0)	101 (19.8)	487 (37.5)	< 0.001
	Try not being outside during evening	674	165 (28.9)	71 (13.9)	438 (33.7)	< 0.001
	Other correct answers	199	56 (9.8)	41 (8.1)	102 (7.8)	0.349
Practice category	Poor	1348	326 (57.2)	364 (71.5)	658 (50.6)	< 0.001
	Good	1031	244 (42.8)	145 (28.5)	642 (49.4)	

^*a*^*p*-values obtained from the chi-square test of independence

### Health-seeking behaviour and malaria case management in children under-five years

The mean health-seeking behaviour score was 3.32 (SD ± 1.34). Overall, around half (662; 53.0%, 95% CI 50.2–55.8%) of the caregivers presented with an adequate level of healthcare-seeking behaviour towards malaria treatment (Table 4). There was no difference in the care-seeking behaviour of caregivers for suspected episodes of malaria among children under-five across the IDPs (167; 51.78%), returnees (136; 48.7%) and host population (359; 55.4%) (p = 0.154). The number of days spent before seeking treatment for a sick child was associated with displacement status with more IDPs (78; 26.4%) spending up to three or more days before seeking malaria care for their feverish under-five children compared to returnees (55; 20.4%) and host (135; 22.0%) (p < 0.001).

**Table 4:** Health seeking behaviour and malaria case management of children under-five in conflict-affected setting of the South West and Littoral Regions of Cameroon

Variables	Category	Total	Study population			p-value^*a*^
			IDPs No (%)	Returnee No (%)	Host No (%)	
Child was ill with a fever at any time in the last two weeks	Yes	1251	323 (72.3)	279 (75.8)	649 (70.4)	0.145
	No	486	124 (27.7)	89 (24.2)	273 (29.6)	
Blood sample during the illness	Yes	726	191 (59.3)	150 (53.8)	385 (59.4)	0.245
	No	523	131 (40.7)	129 (46.2)	263 (40.6)	
Malaria confirmed by a health provider	Yes	812	109 (34.0)	115 (41.5)	205 (31.9)	0.018
	No	429	212 (66.0)	162 (58.5)	438 (69.1)	
Seeks for illness advice/treatment	Yes	1193	299 (92.6)	268 (96.1)	626 (96.5)	0.021
	No	58	24 (7.4)	11 (3.9)	23 (3.5)	
Public medical sector	Government hospital	266	57 (17.6)	43 (15.4)	166 (25.6)	< 0.001
	Government health centre	172	30 (9.3)	42 (15.1)	100 (15.4)	0.026
	Community health worker	457	81 (25.1)	84 (30.1)	218 (33.6)	0.025
Private medical sector	Private hospital	102	37 (11.5)	18 (6.5)	47 (7.2)	0.039
	Private clinic	95	11 (3.4)	15 (5.4)	69 (10.6)	< 0.001
	Pharmacy	228	253 (78.3)	231 (82.8)	539 (83.1)	0.176
NGO medical sector	NGO hospital	39	32 (9.9)	5 (1.8)	2 (0.3)	< 0.001
	NGO clinic	20	14 (4.3)	2 (0.7)	4 (0.6)	< 0.001
Other source	Shop/Market	51	9 (2.8)	14 (5.0)	28 (4.3)	0.350
	Traditional practitioner	38	10 (3.1)	7 (2.5)	21 (3.2)	
	Itinerant drug seller	111	27 (8.4)	33 (11.8)	51 (7.9)	0.139
Number of days treatment was sought for child after the illness began	Same day	222	34 (11.5)	54 (20.4)	134 (21.8)	< 0.001
	One days	273	65 (22.0)	87 (32.8)	121 (19.7)	
	Two days	411	118 (40.0)	69 (26.0)	224 (36.5)	
	Three or more days	268	78 (26.4)	55 (20.8)	135 (22.0)	
Care seeking category	Inadequate	588	156 (48.3)	143 (51.3)	289 (44.6)	0.154
	Adequate	662	167 (51.7)	136 (48.7)	359 (55.4)	

^*a*^*p*-values obtained from the chi-square test of independence

### Malaria treatment practices by caregivers of under-five years children

The mean malaria treatment practice score was 1.24 (SD ± 0.73). The appropriateness of malaria treatment practices for children under-five was low (318; 41.7%, 95% CI 38.2–45.2%) among study participants with no significant difference between IDPs (82; 41.6%), returnees (53; 35.6%) and the host population (183; 43.9%) (p = 0.210). The number of days spent by a sick child before taking anti-malarial medication was significantly associated with displacement status with more IDPs (36; 18.4%) spending more than three days before giving anti-malarial treatment to their feverish under-five children compared to returnees (24; 14.6%) and host (55; 12.1%) (p < 0.001). The malaria treatment practices for the under-five years children are presented on Table 5.

**Table 5:** Malaria treatment practices among children with caregiver-reported fever in conflict-affected setting of the South West and Littoral Regions of Cameroon

Variables	Category	Total	Study population			p-value^*a*^
			IDPs No (%)	Returnee No (%)	Host No (%)	
Medicine taken by the child at any time during the illness	Yes	1154	290 (92.4)	265 (95.7)	599 (95.1)	0.141
	No	67	24 (7.6)	12 (4.3)	31 (4.9)	
Antimalarial medicine taken by the child	ACT	655	178 (55.1)	124 (44.4)	353 (54.4)	0.011
	Fansidar	12	3 (9.9)	6 (2.2)	3 (0.5)	0.053
	Chloroquine	40	13 (4.0)	10 (3.6)	17 (2.6)	0.461
	Amodiaquine	165	40 (12.4)	39 (14.0)	86 (13.3)	0.845
	Quinine injectable	68	16 (5.0)	16 (5.7)	36 (5.5)	0.900
	Artesunate injection	40	9 (2.8)	7 (2.5)	24 (3.7)	0.569
	Artesunate rectal	38	23 (7.1)	3 (1.1)	12 (1.8)	< 0.001
	Other antimalarial	57	14 (4.3)	14 (5.0)	29 (4.5)	0.912
Other medicine	Aspirin	44	6 (1.9)	9 (3.2)	29 (4.5)	0.110
	Paracetamol	535	141 (43.7)	155 (55.6)	239 (36.8)	< 0.001
	Ibuprofen	35	1 (0.3)	12 (4.3)	22 (3.4)	0.005
Number of days spent after the fever before the child first took an ACT	Same day	248	36 (18.4)	44 (26.8)	168 (36.9)	< 0.001
	Next day	286	84 (42.9)	69 (42.1)	133 (29.2)	
	Two days after fever	166	40 (20.4)	27 (16.5)	99 (21.8)	
	More than two days after fever	115	36 (18.4)	24 (14.6)	55 (12.1)	
Malaria treatment practice category	Inappropriate	445	115 (58.4)	96 (64.4)	234 (56.1)	0.210
	Appropriate	318	82 (41.6)	53 (35.6)	183 (43.9)	

ACT: Artemisinin-based combination therapy

^*a*^*p*-values obtained from the chi-square test of independence

### Prevalence of malaria among the children under-five in conflict-affected communities in South West and Littoral Regions

In the course of the community survey, malaria RDT positivity was recorded in 841 (54.5%) of the children under-five (Table 6). The prevalence of malaria in the children under-five was similar across the host (450; 55.8%), returnees (173; 52.0%) and IDP (218; 54.0%) populations (p = 0.473). The Level of asymptomatic malaria was higher among the IDP children (40; 36.4%) compared to their returnee (27; 32.5%) and host counterparts (69; 29.9%), but the difference was not significant (p = 0.484).

**Table 6:** Prevalence of malaria among children under-five in conflict-affected communities of the South West and Littoral Regions of Cameroon

Variables	Category	Total	Study population			p-value^*a*^
			IDPs No (%)	Returnee No (%)	Host No (%)	
Health care provider suspected malaria^b^	Yes	812	212 (65.6)	162 (58.1)	438 (67.6)	0.020
	No	438	111 (34.4)	117 (41.9)	210 (32.4)	
RDT test taken	Yes	1543	404 (90.0)	333 (89.5)	806 (86.6)	0.118
	No	209	45 (10.0)	39 (10.5)	125 (13.4)	
RDT result among tested children	Positive	841	218 (54.0)	173 (52.0)	450 (55.8)	0.473
	Negative	702	186 (46.0)	160 (48.0)	356 (44.2)	
Malaria status						
Symptomatic malaria		705	178 (60.8)	146 (58.9)	381 (66.7)	0.055
Asymptomatic malaria		136	40 (36.4)	27 (32.5)	69 (29.9)	0.484

^a^*p*-values obtained from the chi-square test of independence

^b^Result based on the recall history of the respondents who took their feverish children to a healthcare provider two week prior to the survey

### Factors associated with adequate level of knowledge on malaria prevention

Table 7 presents the results of the fitted multivariate logistic regression model using the enter method to control for age, status in household, educational level, ownership of radio, television and DVD player and the displacement status of the participants. Spouses to the head of household were about 2 times more knowledgeable about malaria prevention compared to other members of the household [AOR 1.55, 95% CI 1.10–2.20, p = 0.012]. The odds of having adequate knowledge on malaria prevention was about 2 times higher in households with radio [AOR 1.49, 95% CI 1.233–1.81, p < 0.001] and television [AOR 1.47, 95% CI 1.18–1.84, p < 0.001], as compared to those without these assets. In comparison to the host population, the returnees showed a 48% chance of reporting with an inadequate level of knowledge towards malaria prevention [AOR 0.52, 95% CI 0.41–0.65, p < 0.001].

**Table 7:** Factors associated with adequate knowledge on malaria prevention among community members in conflict-affected setting of the South West and Littoral Regions of Cameroon

Variable	Category	Level of knowledge		COR	95% CI	p-value	AOR	95% CI	p-value
		Inadequate No (%)	Adequate No (%)						
Age group of caregivers	18–25	175 (53.8)	150 (46.2)	1.00	–	–			
	26–35	348 (50.7)	338 (49.3)	1.13	0.87–1.48	0.354	0.97	0.73–1.28	0.803
	36–45	268 (45.0)	327 (55.0)	1.42	1.09–1.87	0.011	1.17	0.86–1.59	0.317
	46–55	185 (42.0)	255 (58.0)	1.61	1.21–2.15	0.001	1.32	0.94–1.83	0.107
	> 55	143 (44.1)	181 (55.9)	1.48	1.08–2.01	0.013	1.22	0.85–1.76	0.273
Relationship with the head of this household	Head of household	556 (46.0)	654 (54.0)	1.35	0.98–1.86	0.065	1.29	0.92–1.80	0.139
	Spouse	310 (43.7)	400 (56.3)	1.48	1.06–2.07	0.021	1.55	1.10–2.20	0.012
	Son/daughter	162 (56.8)	123 (43.2)	0.87	0.60–1.27	0.478	0.96	0.64–1.42	0.821
	Others	93 (53.4)	81 (46.6)	1.00	–	–			
Highest level of education completed	Informal	55 (53.9)	47 (46.1)	1.00	–	–	1.00	–	–
	Primary	550 (49.5)	560 (50.5)	1.19	0.79–1.79	0.339	0.65	0.39–1.08	0.095
	Secondary	439 (45.0)	537 (55.0)	1.43	0.95–2.16	0.086	0.75	0.54–1.03	0.079
	Tertiary	77 (40.3)	114 (59.7)	1.73	1.07–2.81	0.026	0.87	0.63–1.20	0.391
Type of available household assets									
Radio	No	632 (55.6)	504 (44.4)	1.00	–	–	1.00	–	–
	Yes	445 (39.0)	697 (61.0)	1.96	1.66–2.32	0.000	1.58	1.33–1.89	< 0.001
Television	No	460 (58.4)	327 (41.6)	1.00	–	–	1.00	–	–
	Yes	616 (40.6)	902 (59.4)	2.06	1.73–2.45	0.000	1.76	1.44–2.14	< 0.001
DVD player	No	822 (49.9)	825 (50.1)	1.00	–	–			
	Yes	299 (40.8)	433 (59.2)	1.44	1.21–1.72	0.000	1.02	0.83–1.24	
Displacement status	IDPs	288 (50.5)	282 (49.5)	0.67	0.55–0.81	0.000	0.87	0.69–1.08	0.199
	Returnee	307 (60.3)	202 (39.7)	0.45	0.36–0.55	0.000	0.52	0.41–0.65	< 0.001
	Host	526 (40.5)	774 (59.5)	1.00	–	–	1.00	–	–

## Discussion

In this study, the knowledge, attitudes and practices for malaria prevention among host, returnees and IDP populations were explored in a cross-section of communities in the SW and Littoral Regions of Cameroon affected by the ongoing armed-conflict. Furthermore, the healthcare-seeking behaviour of caregivers of under-five years children relating to episodes of suspected malaria was explored together with the prevalence of malaria among these children. The baseline findings presented in this study are important to inform the design of malaria control programmes and for the identification of barriers to effective malaria treatments in this conflict setting.

The results of this study showed that a little over half of the community presented with adequate knowledge about malaria prevention and this was even lower among the IDPs and returnee population. Almost all the respondents associated the transmission of malaria with mosquito bites, which is consistent with results from similar studies conducted in malaria endemic areas [11–13]. This association also infers that, preventive interventions targeted at reducing exposure to mosquitoes bites may be effective, given this baseline knowledge of transmission. The level of knowledge concerning malaria prevention was associated to the ownership of television and radio among the study participants. These communities are located in malaria endemic zones which might have favored communication about the disease through educational talks on the radio, television channels or community health workers. The symptoms of malaria most frequently cited by respondents in the current study were fever and headache, which are consistent with studies conducted in Swaziland [14] and South Africa [11]. The current findings reveal that the community lack comprehensive knowledge of malaria symptoms (Table 2). This is an important observation as knowledge of signs and symptoms play a particular role in healthcare seeking, early diagnosis and treatment of malaria [15].

The majority of the participants knew that they could seek help when any malaria symptoms are present but less than half of them cited the CHWs as persons to contact for assistance. This could suggest that community members are not familiar with the presence and role of CHWs in their communities or indicate a lack of confidence in CHW’s capacity to serve as their first line of contact in accessing malaria care and treatment at the primary healthcare level. The host population were better informed that they could seek for help from the health facility whenever faced with fever, which represents a potential barrier for the IDPs and returnees to access services at the hospital due to lack of knowledge on seeking healthcare from the hospital. The community’ knowledge of the test to diagnose malaria was below 50%. Moreover, 37.2% of the respondents were not aware that they were supposed to request for a rapid diagnostic test to diagnose malaria before being treated for the disease. The majority of the participants were quite aware that malaria poses a risk to their health and that the disease can cause long-term consequences. This may be the reason for a common practice of self-medication in these communities where drugs are consumed presumptively once fever is present or malaria is suspected [16]. Most households practiced at least one malaria prevention method; mosquito bed nets were the most frequently used measure. This finding is consistent with a similar study conducted in Ethiopia where the majority of respondents considered the mosquito bed net as a protective measure against mosquito bites [17]. Mosquito bed nets are a key part of the national malaria prevention control programme in Cameroon with a national coverage of 59.0% out of a target of 80.0% [10]. In the SW Region 46.0% of the households have access to mosquito bed nets [10]. Despite the widely reported ownership of bed nets in the accessible areas of the conflict-affected communities, there is lack of data to show that members of households sleep under the nets as this was not captured in the current study. The armed conflict has disrupted the distribution of bed nets and those remaining in the community may have experienced wear and damage over time. The last distribution of long-lasting insecticidal nets (LLINs) was in 2017 and a distribution was planned for 2022. This could also be as a result that communities in the SW region were yet to receive bed nets from the planned mass distribution campaign supported by the Global Fund to fight AIDS, Tuberculosis and Malaria, due to the insecurity in the region. Malaria prevention methods were poorly implemented across these communities as less than 50.0% of the surveyed households presented with adequate level of practices aimed at preventing malaria. This was lower for households of IDPs and returnees as they might have left behind these protective methods or lost them along the way in the course of their displacement from their usual habitation. Promoting community participation [18] and encouraging behaviour change may contribute to the fight against malaria in these settings.

These findings clearly demonstrate that malaria is a common community problem as evident by majority of the caregivers attesting their children were ill with fever in the last 2 weeks prior to the survey. Furthermore, the majority of children with fever were confirmed by health workers to be suffering from malaria after conducting an RDT. The study recorded a high RDT positivity rate of 54.5% (Table 6). The finding of the current study is higher than the 24.0% national malaria prevalence recorded for children 6–59 months in 2018 in Cameroon [10]. The national figure of 24.0% was based on health facility data and could have underestimated the actual burden in the community captured by the current community-based assessment. The high burden of malaria in the conflict-affected communities might be due to the difficult riverine and tropical environment together with disruption of malaria prevention and care services resulting from the destruction of primary care facilities, precarious living conductions of IDPs and inaccessibility of the host and IDPs population to treatment services. Moreover, this formative research was conducted in the peak of malaria transmission in the conflict-affected settings to develop community-led approaches tailored for the control malaria.

The health-seeking behaviours towards suspected episodes of malaria demonstrated by the caregivers of children under-five in this study was low, only 53.0% had adequate levels of care seeking for their children. A positive finding was that a good proportion of the population preferred to seek health care for their children from the CHWs in the community in additional to the preference for health staff in the hospitals although less than 50.0% of children reported with fever were treated within the first 24 h of onset of symptoms. Seeking treatment following an episode of fever on the same day or one day later was observed to be practiced by very few of the caregivers while most of them stay with the sick child for up to three or more days. The observed delay in seeking care for feverish children was higher for the IDP population compared to their host counterparts. This observation might be associated with financial barriers plaguing these communities whose sources of livelihood had been disrupted as a result of the ongoing conflict.

In the multivariate analysis, an association was observed between the ownership of radio and television and knowledge about malaria prevention. Access to malaria information through the radio and television has been observed from mass media implementation of malaria prevention measures in sub-Saharan African countries [19]. This is likely to be disrupted as the conflict progresses with the potential of more frequent displacements. Empowering these communities with correct knowledge on malaria prevention and to adopt better health-seeking behaviours could be achieved through community organized efforts [18].

### Strengths and limitations of the study

The large sample size, generalizability of the findings to armed conflict setting characteristic of the inclusion of different population groups are among the strengths of this study. Children were tested for malaria using RDT which is more accurate than self-reported episodes of fever. However, positive RDTs were considered diagnostic of malaria irrespective of whether or not the child had been treated for fever within the past one month. This could increase the rate of false positives as antibodies to the parasites could have been wrongly classified an active malaria infection. Moreover, the community experienced several episodes of displacement following active fighting between armed groups whose frequency were not captured. This displacement further exposes the respondents and their children to mosquito bites. This could have confounded the exact burden of malaria among children under five in this present study. The findings of this study will enable Reach Out and its partners to design and implement community-based malaria services targeted at fostering community participation to improve access to malaria prevention and treatment.

## Conclusion

Over half of the community members know the methods to prevent malaria with the use of mosquito bed nets being utilized as the main preventive measure. Health-seeking behaviours are sub-optimal and there is a high prevalence of malaria among children under-five. Overall, the results shown in this study raises the need to increase access to information that will enhance practices on malaria prevention and health-seeking and also to improve the quality of malaria treatment services that are sought by the population. Furthermore, the services provided by the formal health care system through the CHWs and health facilities should be well coordinated to break barriers hindering the uptake of malaria services by the host and IDPs populations. The findings of this study have inspired the design of three interventions to break barriers in access to effective malaria treatment services for host and displaced populations in the SW and Littoral Regions. An adapted community dialogue approach termed “Community Health Participatory Approach”, is being implemented to increase community knowledge, attitudes and practices, and improve healthcare-seeking behaviours. Health vouchers are provided to support severe malaria treatment for children under-five and covering transport costs to access health facilities whereas simple malaria is treated using CHWs. The CHWs are supportively supervised to enhance the quality of malaria services provided to the local population. These interventions are currently being implemented in 64 and 16 communities in the SW and Littoral Regions respectively. The findings will contribute to informing the strategies of the National Malaria Control Programme in the Ministry of Public Health and the mandate of the Global Fund to fight malaria. Conflict-affected settings should be actively engaged in the efforts to eliminate malaria as a public health problem.

### Supplementary Information

**Additional file 1.** Paper-based structured questionnaire used for baseline data collection.

## Data Availability

The data used for this study are available from the corresponding author on reasonable request.
