# Supplementary material for: A formative cross-sectional study to assess caregiver’s health-seeking behaviour and knowledge surrounding malaria, and understand the burden of malaria among children under-five in conflict-affected communities of Cameroon
Source: Malar J. 2024 Apr 8;23:99. doi: 10.1186/s12936-024-04902-5 (PMC11003128; doi:10.1186/s12936-024-04902-5)
Supplement: Supplementary file 1 — Additional file 1. Paper-based structured questionnaire used for baseline data collection. [file 12936_2024_4902_MOESM1_ESM.pdf]

Research study: Breaking barriers in access to effective malaria treatment among conflict-affected communities

KAP Survey – Malaria care among conflict-affected populations

Section 1: Identification

**Cluster code:** ☐☐	**Household code:** ☐☐
**Interviewer ID:** ☐☐	**Date: (dd/mm/yy)** ______/07/14
**Start time:**	**Respondent's gender:** ☐☐<br>01 = Male<br>02 = Female

Section 2: Interviewee and Household Details

	QUESTIONS AND INSTRUCTIONS	CODING CATEGORIES	SKIP
**Q1**	**How old are you?**<br><br>SINGLE RESPONSE	0-17 years........01<br>18-25 years........02<br>26-35 years........03<br>36-45 years........04<br>46-55 years........05<br>56-65 years........06<br>Over 65 years........07<br><br>Don't know........88<br>No answer........99	
**Q2**	**What is your religion?**<br><br>SINGLE RESPONSE	None........01<br>Christian (Protestant)........02<br>Christian (Roman Catholic)........03<br>Christian (Pentecostal)........04<br>Christian (Seventh Day Adventist)........05<br>Muslim........06<br>Hindu........07<br>Other........08<br><br>Specify:________________________________<br><br>Don't know........88<br>No answer........99	

	QUESTIONS AND INSTRUCTIONS	CODING CATEGORIES	SKIP
Q3	**What is your relationship with the head of this household?**<br><br>SINGLE RESPONSE	Head of household .... 01<br>Spouse .... 02<br>Son/daughter .... 03<br>Stepchild .... 04<br>Adopted/foster child .... 05<br>Son-in-law/daughter-in-law .... 06<br>Grandchild .... 07<br>Parent .... 08<br>Stepparent .... 09<br>Parent-in-law .... 10<br>Brother/sister .... 11<br>Brother-in-law/sister-in-law .... 12<br>Nephew/niece .... 13<br>No relation .... 14<br>Other .... 15<br><br>Specify:________________________________<br><br>Don't know .... 88<br>No answer 99	
Q4	**What is your main occupation?**<br><br>SINGLE RESPONSE	None .... 01<br>Public sector employee (professional) .... 02<br>Public sector employee (manual) .... 03<br>Private sector employee (professional) .... 04<br>Private sector employee (manual) .... 05<br>Self-employed (business) .... 06<br>Self-employed (agriculture/fishery) .... 07<br>Domestic work in household .... 08<br>Religious leader .... 09<br>Teacher .... 10<br>Student .... 11<br>Jobless .... 12<br>Incapacitated .... 13<br>Other .... 14<br><br>Specify:________________________________<br><br>Don't know .... 88<br>No answer 99	

	QUESTIONS AND INSTRUCTIONS	CODING CATEGORIES	SKIP
**Q5**	**What is the highest level of education you have completed?**<br><br>SINGLE RESPONSE	None .... 01<br>Started but did not complete primary .... 02<br>Primary .... 03<br>Secondary .... 04<br>*Médio* .... 05<br>Technical course – short duration .... 06<br>Professional course .... 07<br>University .... 08<br>Other .... 12<br><br>Specify:____________________<br><br>Don’t know .... 88<br>No answer 99	
**Q6**	**Does your household have any of the following assets?**<br><br>ASK ABOUT EACH OF THE ASSESTS AND CIRCLE "YES" IF THEY OWN IT OR "NO" IF THEY DO NOT	Yes / No / Don’t know / No answer<br><br>Radio .... 01 02 88 99<br>Television .... 01 02 88 99<br>DVD player .... 01 02 88 99<br>Mobile<br>phone/landlin 01 02 88 99<br>e...................... 01 02 88 99<br>Motor bike........	
**Q7**	**What sources of water does your household use, for example for drinking or washing clothes?**<br><br>MULTIPLE RESPONSES POSSIBLE<br>CIRCLE ALL MENTIONED<br><br>PROBE TWICE: ANYTHING ELSE?	Water channeled from house to house .... 01<br>Public fountain .... 02<br>Unprotected well .... 03<br>Protected well .... 04<br>Unprotected fountain .... 05<br>Protected fountain .... 06<br>Bore hole .... 07<br>River .... 08<br>Lagoon .... 09<br>Rain water .... 10<br>Bottled water .... 11<br>Tanker-truck .... 12<br>Cistern .... 13<br>Other .... 14<br><br>Specify:____________________<br><br>Don’t know .... 88<br>No answer 99	

	QUESTIONS AND INSTRUCTIONS	CODING CATEGORIES	SKIP
Q8	**What kind of toilet facility do members of your household typically use?**<br><br>SINGLE RESPONSE	Pit latrine .... 01<br>Conventional latrine .... 02<br>Improved latrine .... 03<br>Bush/field .... 04<br>Other .... 05<br><br>Specify:___________________<br><br>Don't know .... 88<br>No answer 99	
Q9	**Displacement status**<br>**Internally displaced persons** (**IDPs**): people who have been forced or obliged to flee their homes or places of habitual residence, in order to avoid the effects of armed conflict or violations of human rights and who have not crossed an internationally recognized state border"<br>**Returnee:** A person who was displaced, but has recently returned to his/her village or town of origin.<br>**Non-displaced person**: A person still living in their village/town of origin since the start of the crisis.	**IDP = 01 = Yes**<br>**02 = No** ☐☐<br>If Yes<br>Village/town of Origin_______________<br><br>Division of Origin ___________________<br><br>Region of Origin____________________<br><br>**Returnee = 01 = Yes**<br>**02 = No** ☐☐<br>If Yes<br>Village/town of Origin__________________<br><br>Division of Origin______________________<br><br>Region of Origin _______________________<br><br>**Non-displaced = 01 = Yes**<br>**02 = No** ☐☐	

Section 3: Malaria

	QUESTIONS AND FILTERS	CODING CATEGORIES	SKIP
Q10	**Have you heard of malaria?**<br><br>SINGLE RESPONSE	Yes .... 01<br>No .... 02<br><br>Don't know .... 88<br>No answer 99	 → END<br><br>→ END<br>→ END

	QUESTIONS AND FILTERS	CODING CATEGORIES	SKIP
**Q11**	**In the last six months, where did you hear about it?**<br><br>MULTIPLE RESPONSES POSSIBLE<br>CIRCLE ALL MENTIONED<br><br>PROBE TWICE: ANYTHING ELSE?	Not heard about malaria in the last six months .... 01<br>Health professional .... 02<br>Community health worker .... 03<br>Community meeting........ 04<br>School........ 05<br>Radio/TV........ 06<br>Newspaper .... 07<br>Political leader........ 08<br>Relative/friend/neighbour .... 09<br>Poster .... 10<br>Other .... 11<br><br>Specify:___________________<br><br>Don't know........ 88<br>No answer 99	
**Q12**	**How do you get malaria?**<br><br>MULTIPLE RESPONSES POSSIBLE<br>CIRCLE ALL MENTIONED<br><br>PROBE TWICE: ANYTHING ELSE?	Mosquito bite........ 01<br>Other .... 02<br><br>Specify:___________________<br><br>Don't know........ 88<br>No answer 99	
**Q13**	**Do you know how you can avoid getting malaria?**<br><br>MULTIPLE RESPONSES POSSIBLE<br>CIRCLE ALL MENTIONED<br><br>PROBE ONCE: ANYTHING ELSE?	Use a mosquito net .... 01<br>Avoid being outdoors at night time .... 02<br>Others........ 03<br><br>Specify:___________________<br><br>Don't know........ 88<br>No answer 99	

	QUESTIONS AND FILTERS	CODING CATEGORIES	SKIP
**Q14**	**What are the possible symptoms of malaria?**<br><br>MULTIPLE RESPONSES POSSIBLE<br>CIRCLE ALL MENTIONED<br><br>PROBE ONCE: ANYTHING ELSE?	Fever.......1<br>Headache.......2<br>Body aches.......3<br>Blood in urine.......4<br>Diarrhoea.......5<br>Nausea/vomiting.......6<br>Rash/itch.......7<br>Weight loss.......8<br>Other.......9<br><br>Specify:___________________________<br><br>Don't know.......88<br>No answer.......99	 <br><br><br><br><br><br><br><br><br><br><br><br><br><br><br>➔ Q19<br>➔ Q19
**Q15**	**If you have any of these symptoms, would you seek help?**<br><br>SINGLE RESPONSE	Yes.......01<br>No.......02<br><br>Don't know.......88<br>No answer.......99	 ➔ Q18<br><br>➔ Q19<br>➔ Q19
**Q16**	**Who would you ask for help?**<br><br>MULTIPLE RESPONSES POSSIBLE<br>CIRCLE ALL MENTIONED<br><br>PROBE ONCE: ANYONE ELSE?	Community leader.......01<br>Traditional healer.......02<br>Pharmacy/drug vendor.......03<br>Community health worker.......04<br>Health worker/health facility.......05<br>Family member.......06<br>Other.......07<br><br>Specify:___________________________<br><br>Don't know.......88<br>No answer.......99	➔ Q19<br>➔ Q19<br>➔ Q19<br>➔ Q19<br>➔ Q19<br>➔ Q19<br>➔ Q19<br>➔ Q19<br>➔ Q19<br>➔ Q19<br><br><br><br>➔ Q19<br>➔ Q19

	QUESTIONS AND FILTERS	CODING CATEGORIES	SKIP
**Q17**	**Why not?**<br><br>MULTIPLE RESPONSES POSSIBLE<br>CIRCLE ALL MENTIONED<br><br>PROBE ONCE: ANY OTHER REASON?	I don't have anyone to go to .... 01<br>I have no money .... 02<br>I am not concerned about the symptoms .... 03<br>I never seek medical help .... 04<br>Other .... 05<br><br>Specify:____________________<br><br>Don't know .... 88<br>No answer 99	
**Q18**	**Do you know the test for malaria?**<br><br>SINGLE RESPONSE<br><br>IF RESPONDENT DOES NOT KNOW THE NAME OF THE DRUG, SAY "THE TEST USED TO TREAT MALARIA IS CALLED AN RDT"	Yes, I know the name .... 01<br>No, I don't know the name .... 02<br><br>Don't know .... 88<br>No answer 99	 ➔ Q22<br><br>➔ Q22<br>➔ Q22
**Q19**	**Do you always ask for an RDT before being treated for malaria?**<br><br>SINGLE RESPONSE	Yes, ... 01<br>No .... 02<br><br>Don't know .... 88<br>No answer 99	 ➔ Q22<br><br>➔ Q22<br>➔ Q22
**Q20**	**Do you know the name of the drug to treat malaria?**<br><br>SINGLE RESPONSE<br><br>IF RESPONDENT DOES NOT KNOW THE NAME OF THE DRUG, SAY "THE DRUG USED TO TREAT MALARIA IS CALLED COARTEM"	Yes, I know the name .... 01<br>No, I don't know the name .... 02<br><br>Don't know .... 88<br>No answer 99	 ➔ Q22<br><br>➔ Q22<br>➔ Q22
**Q21**	**How did you feel after taking the medication?**<br><br>SINGLE RESPONSE	Felt fine .... 01<br>Felt slightly unwell .... 02<br>Felt very unwell .... 03<br><br>Don't know .... 88<br>No answer 99	

	QUESTIONS AND FILTERS	CODING CATEGORIES	SKIP
**Q22**	**Do you agree with the following statements?**<br><br>**My household is affected by malaria.**<br><br>**Malaria is a matter of concern for me.**<br><br>**Malaria can have long-term consequences for my health.**<br><br>**It is important to have a test before taking malaria treatment**<br><br>**Coartem is the best cure for malaria.**<br><br>CIRCLE RESPONDENT'S REPLY FOR EACH OF THE STATEMENTS.	Yes / No / Don't know / No answer<br><br>01 02 88 99<br><br>01 02 88 99<br><br>01 02 88 99<br><br>01 02 88 99<br><br>01 02 88 88	
**Q23**	**Do you do anything to protect you and your household from malaria?**<br><br>SINGLE RESPONSE	Yes .... 01<br>No .... 02<br><br>Don't know .... 88<br>No answer 99	 → Q40<br><br>→ END<br>→ END
**Q24**	**What do you do?**<br><br>MULTIPLE RESPONSES POSSIBLE<br>CIRCLE ALL MENTIONED<br><br>PROBE ONCE: ANYTHING ELSE?	Sleep under a mosquito net .... 01<br>Use netting on windows .... 02<br>Try not to be outside during evening .... 03<br>Other .... 04<br><br>Specify:_______________<br><br>Don't know .... 88<br>No answer 99	→ END<br>→ END<br>→ END<br>→ END<br>→ END<br><br>→ END<br>→ END<br>→ END<br><br>→ END<br>→ END
**Q25**	**Why not?**<br><br>MULTIPLE RESPONSES POSSIBLE<br>CIRCLE ALL MENTIONED<br><br>PROBE ONCE: ANY OTHER REASON?	Don't know what I can do .... 01<br>I'm not concerned about malaria .... 02<br>I don't have money .... 03<br>It is not practical .... 04<br>Other .... 05<br><br>Specify:_______________<br><br>Don't know .... 88<br>No answer 99	

Section 4: Malariometric Section

(From https://www.dhsprogram.com/publications/publication-MISQ8-MIS-Questionnaires-and-Manuals.cfm - will be adapted for Cameroon context)

NO.	QUESTIONS AND FILTERS	CODING CATEGORIES	SKIP
401	CHECK 216, 217, AND 218 IN THE BIRTH HISTORY: ANY SURVIVING CHILDREN BORN 0-59 MONTHS BEFORE THE SURVEY?<br>ONE OR MORE SURVIVING CHILDREN BORN 0-59 MONTHS BEFORE THE SURVEY / NO SURVIVING CHILDREN BORN 0-59 MONTHS BEFORE THE SURVEY		417
402	Now I would like to ask some questions about the health of your children born in the last 5 years. (We will talk about each separately, starting with the youngest.)		
403	RECORD THE NAME AND BIRTH HISTORY NUMBER FROM 213 OF THE SURVIVING CHILDREN BORN 0-59 MONTHS BEFORE THE SURVEY, STARTING WITH THE LAST ONE.<br>NAME OF CHILD	BIRTH HISTORY NUMBER	
404	Has (NAME) been ill with a fever at any time in the last 2 weeks?	YES . . . . 1<br>NO . . . . 2<br>DON'T KNOW 8	416
405	At any time during the illness, did (NAME) have blood taken from (NAME)'s finger or heel for testing?	YES . . . . 1<br>NO . . . . 2<br>DON'T KNOW 8	
406	Were you told by a healthcare provider that (NAME) had malaria?	YES . . . . 1<br>NO . . . . 2<br>DON'T KNOW 8	
407	Did you seek advice or treatment for the illness from any source?	YES . . . . 1<br>NO 2	412

NO.	NAME OF CHILD ______________________	BIRTH HISTORY NUMBER . . . . [ ][ ]	
408 (1)	Where did you seek advice or treatment?<br><br>Anywhere else?<br><br>PROBE TO IDENTIFY THE TYPE OF SOURCE.<br><br>IF UNABLE TO DETERMINE IF PUBLIC, PRIVATE, OR NGO SECTOR, RECORD 'X' AND WRITE THE NAME OF THE PLACE(S).	**PUBLIC SECTOR**<br>GOVERNMENT HOSPITAL .... A<br>GOVERNMENT HEALTH CENTER .... B<br>GOVERNMENT HEALTH POST .... C<br>MOBILE CLINIC .... D<br>COMMUNITY HEALTH WORKER/<br>FIELDWORKER .... E<br>OTHER PUBLIC SECTOR<br>SECTOR ______________________ F<br>(SPECIFY)<br><br>**PRIVATE MEDICAL SECTOR**<br>PRIVATE HOSPITAL .... G<br>PRIVATE CLINIC .... H<br>PHARMACY .... I<br>PRIVATE DOCTOR .... J<br>MOBILE CLINIC .... K<br>COMMUNITY HEALTH WORKER/<br>FIELDWORKER .... L<br>OTHER PRIVATE MEDICAL<br>SECTOR ______________________ M<br>(SPECIFY)<br><br>**NGO MEDICAL SECTOR**<br>NGO HOSPITAL .... N<br>NGO CLINIC .... O<br>OTHER NGO MEDICAL<br>SECTOR ______________________ P<br>(SPECIFY)<br><br>**OTHER SOURCE**<br>SHOP .... Q<br>TRADITIONAL PRACTITIONER .... R<br>MARKET .... S<br>ITINERANT DRUG SELLER .... T<br><br>OTHER ______________________ X<br>(SPECIFY)	
409	CHECK 408: TWO OR MORE CODES CIRCLED [ ] ↓	ONLY ONE CODE CIRCLED [ ]	→ 411
410	Where did you first seek advice or treatment?<br><br>USE LETTER CODE FROM 408	FIRST PLACE . . . . [ ]	
411	How many days after the illness began did you first seek advice or treatment for (NAME)?<br><br>IF THE SAME DAY RECORD '00'.	DAYS . . . . [ ][ ]	
412	At any time during the illness, did (NAME) take any medicine for the illness?	YES .... 1<br>NO .... 2<br>DON'T KNOW 8	→ 416

NO.	NAME OF CHILD	BIRTH HISTORY NUMBER	
413 (2)	What medicine did (NAME) take?<br><br>Any other medicine?<br><br>RECORD ALL MENTIONED.<br><br>IF MEDICINE NOT KNOWN, ASK TO SEE THE PACKAGE OR PRESCRIPTION.	**ANTIMALARIAL MEDICINE**<br>ARTEMISININ COMBINATION<br>THERAPY (ACT) . . . . A<br>SP/FANSIDAR . . . . B<br>CHLOROQUINE . . . . C<br>AMODIAQUINE . . . . D<br>QUININE<br>PILLS . . . . E<br>INJECTION/IV . . . . F<br>ARTESUNATE<br>RECTAL . . . . G<br>INJECTION/IV . . . . H<br><br>OTHER<br>ANTIMALARIAL______________________________I<br>(SPECIFY)<br><br>**ANTIBIOTIC MEDICINE**<br>AMOXICILLIN . . . . J<br>COTRIMOXAZOLE . . . . K<br>OTHER PILL/SYRUP . . . . L<br>OTHER INJECTION/IV . . . . M<br><br>**OTHER MEDICINE**<br>ASPIRIN . . . . N<br>PARACETAMOL/PANADOL/ ACETAMINOPHEN . . . .<br>. . . . . O<br>IBUPROFEN . . . . P<br><br>OTHER______________________________________X<br>(SPECIFY)<br><br>DON'T KNOW . . . . Z	
414	CHECK 413: ARTEMISININ COMBINATION THERAPY ('A') GIVEN<br><br>CODE 'A' CIRCLED	CODE 'A' NOT CIRCLED	416
415	How long after the fever started did (NAME) first take an artemisinin combination therapy?	SAME DAY . . . . 0<br>NEXT DAY . . . . 1<br>TWO DAYS AFTER FEVER . . . . 2<br>THREE OR MORE DAYSAFTER FEVER . . . . . 3<br>DON'T KNOW 8	
416	CHECK 216 AND 217 IN BIRTH HISTORY: ANY MORE SURVIVING CHILDREN BORN 0-59 MONTHS BEFORE THE SURVEY?<br><br>NO MORE SURVIVING CHILDREN BORN 0-59 MONTHS BEFORE THE SURVEY	MORE SURVIVING CHILDREN BORN 0-59 MONTHS BEFORE THE SURVEY	403

417	RECORD THE TIME.	HOURS ....<br>MINUTES	

END

DO YOU HAVE ANY QUESTIONS?

THANK YOU FOR YOUR TIME.

Section 5: Final Result

TO BE COMPLETED BY THE FIELD RESEARCHER

**End time:**	**Final result code:** ☐☐<br><br>01 = Completed<br>02 = Partially completed

Section 5: Post-interview
TO BE COMPLETED BY SUPERVISOR
Supervisor code: ☐☐

Supervisor's signature:
